# Demographic Profile and Comorbidities Among Cataract Patients at a Tertiary Hospital in Visakhapatnam

**DOI:** 10.7759/cureus.85007

**Published:** 2025-05-28

**Authors:** Narsimha Rao Arepalli, Anika Goel, Rahul Dharma Teja Gangavarapu, Venkata Dileep Kumar Veldi, Dhwani A Mehta, Anirudh Srinivas Teja Peela, Prem Chemudugunta, Swarada Joshi, Sri Sai Praneeth Angara

**Affiliations:** 1 Ophthalmology, Gayatri Vidhya Parishad Institute of Healthcare and Medical Technology, Visakhapatnam, IND; 2 Medicine, Kakatiya Medical College, Warangal, IND; 3 Medicine, Gayatri Vidhya Parishad Institute of Healthcare and Medical Technology, Visakhapatnam, IND; 4 Medicine, NRI Institute of Medical Sciences, Visakhapatnam, IND; 5 Ophthalmology, NRI Institute of Medical Sciences, Visakhapatnam, IND

**Keywords:** blindness, cataract, comorbidities, diabetes mellitus, disease severity, hypertension, ophthalmology, public health, tertiary care hospital, visual impairment

## Abstract

Introduction: Cataract is the leading cause of preventable blindness worldwide, with a particularly high burden in developing countries. Despite advancements in surgical interventions, barriers such as limited healthcare access, socioeconomic disparities, and comorbid systemic conditions contribute to disease progression and severity. This study aims to analyze the demographic profile of cataract patients and the prevalence of comorbidities affecting disease severity in a tertiary care hospital in Visakhapatnam.

Methodology: A cross-sectional, hospital-based observational study was conducted at Gayatri Vidya Parishad Institute of Healthcare and Medical Technology (GVPIHC&MT), Visakhapatnam, over a period of two months from October to November 2024. A total of 105 cataract patients were included based on the inclusion criteria. Data were collected using a structured proforma covering clinicodemographic characteristics, cataract type (LOC-III), and systemic comorbidities. Cataract severity was graded into four levels based on the slit-lamp examination of cataract patients. Descriptive statistics and chi-square tests were employed for data analysis, with statistical significance set at p < 0.05.

Results: Among the 105 participants, 55 (52.38%) were aged above 60 years, and 58 (55.24%) were female. The majority, 72 (68.57%), belonged to the middle socioeconomic class, with 43 (40.95%) residing in urban areas. Mixed cataracts were the most prevalent type, found in 74 (70.48%) cases, and 38 (36.19%) cases were categorized as very severe. The most common comorbidities were hypertension, affecting 43 (40.95%) participants, and diabetes mellitus, affecting 35 (33.33%), both of which were significantly associated with cataract severity (p = 0.01 and p = 0.03, respectively). The duration of symptoms varied, with 52 (49.52%) participants reporting symptoms persisting for 12 months.

Conclusion: This study highlights the substantial burden of cataracts in older adults, with significant associations between systemic comorbidities and disease severity. Hypertension and diabetes mellitus were found to be key contributors to advanced cataract stages, emphasizing the need for integrated healthcare approaches addressing both ocular and systemic health. Targeted screening and early intervention strategies are essential to reduce cataract-related morbidity, particularly in vulnerable populations with limited healthcare access.

## Introduction

Cataract is the most common cause of preventable blindness globally, and it is dominant in developing countries. It is responsible for nearly three-quarters of the global burden of cataract. The Blue Mountains Eye Study (BMES) revealed that 72% of participants developed cataracts in one or both eyes over a 10-year period, highlighting its prevalence and implications for public health [1].

Women have a greater burden of cataract worldwide than men, predominantly because of differences in life expectancy and risk exposures. In addition, the majority of cataracts are attributed to ageing, so it is a disease tightly related to demographic transitions that are taking place in most populations [2].

In India, cataracts account for 62.6% of all blindness, affecting an estimated 9-12 million bilaterally blind individuals. The World Health Organization (WHO) reports that cataracts account for 47.8% of blindness and are a focus of blindness prevention project [3]. Despite the improvements of surgical intervention, cataract blindness still contributes to high rates in developing nations mainly because of shortcomings in the healthcare infrastructure, lack of knowledge, and poverty.

Socioeconomic factors further exacerbate the burden of cataracts. Research such as the Baltimore Eye Survey and the Singapore Malay Eye Study have shown significant associations between low socioeconomic status (SES) and prevalence of cortical, nuclear, and posterior subcapsular cataracts [4]. 

Considering these limitations, while several studies have explored the burden of cataracts globally, there is limited data on the demographic and comorbid profile of cataract patients in Visakhapatnam, India. The present study aims to bridge this gap by investigating the demographic profile of cataract patients and the burden of comorbid conditions among those visiting a tertiary care center in Visakhapatnam. By examining these patterns, this research seeks to inform targeted interventions and improve outcomes for this vulnerable population. Furthermore, this study aims to assess the demographic characteristics of cataract patients and evaluate the influence of comorbid systemic disorders on cataract severity in a tertiary care hospital.

## Materials and methods

Materials

This observational, cross-sectional, hospital-based study was conducted from October to November 2024 at the Gayatri Vidya Parishad Institute of Healthcare and Medical Technology (GVPIHC&MT), located in Visakhapatnam, India. The study population comprised patients with cataracts attending the tertiary care facility in the Visakhapatnam district. Data collection focused on demographic factors, clinical characteristics of the cataracts, and patient-reported outcomes. The duration of the study spanned two months, aiming to analyze the prevalence and impact of cataract severity on daily living and visual function among the patients.

Sample Size

A total of 121 patients who were diagnosed with cataract in a respective tertiary care hospital, among them, 105 patients were included in the current study. This sample size was determined based on a convenience sampling technique, considering the hospital's patient output and the expected prevalence of cataract among patients in Visakhapatnam.

Study Tool

A structured case study form was used to assess the prevalence of cataract (Appendix 1,2).

Inclusion Criteria

All patients who are having cataracts and who agreed to written voluntary informed consent were considered for participation in the study after a thorough explanation of the study.

Exclusion Criteria

Patients having other ocular diseases affecting cataract, like uveitis (Fuchs heterochromic iridocyclitis), glaucoma, and retinal diseases like retinal pigmentosa, diabetic retinopathy, and hypertensive retinopathy.

Method

The clinicodemographic data with regard to age, sex, gender, socioeconomic status, cataract-affected eye, duration and type of cataract, and other systemic comorbidities were collected in a well-designed proforma. Cataract severity was based on nucleus hardness on slit-lamp biomicroscopy and was graded from I to IV, where Grade I is soft with a white or greenish-yellow nucleus, Grade II is soft-medium with a yellowish hue, Grade III is medium-hard with an amber color, Grade IV is hard with a brownish nucleus, and Grade IV is ultrahard (rock hard) with a blackish appearance. Cataract type was classified based on the Lens Opacities Classification System III (LOCS III) as nuclear, cortical, posterior subcapsular, and mixed types.

Data Collection Procedures

Data was collected using a pretested, structured proforma designed to capture comprehensive clinicodemographic information from participants. This included demographic details such as age, sex, and gender, along with socioeconomic status. The proforma also gathered specific information regarding cataracts, including the affected eye, duration, type, and severity of the condition. Additionally, it documented any associated systemic comorbidities, such as diabetes and hypertension. To evaluate the severity of cataracts, each case was graded into one of four categories, ranging from grade I to grade IV. This structured approach ensured systematic and reliable data collection for analysis.

Ethical Considerations

Approval was taken from the institution's IEC before commencing the study (Ref. No.: GVPIHCMT/IEC/20240917/10). Participants were fully informed about the study’s purpose and procedures before providing written consent. Their involvement was entirely voluntary, with the freedom to withdraw at any time without consequences. To ensure confidentiality, unique identification codes replaced personal details in all reports. The study posed minimal risk, and support services, including counseling and referrals, were available for anyone experiencing discomfort. Regular checks were performed to ensure quality control.

Data Analysis

Data were analyzed using both descriptive and inferential statistics. Clinicodemographic variables, including age, gender, socioeconomic status, and cataract characteristics, were summarized using descriptive statistics. To examine associations between cataract severity, demographic factors, and comorbidities, chi-square tests were applied. All statistical analyses were conducted using IBM SPSS Statistics for Windows, Version 26 (Released 2019; IBM Corp., Armonk, New York, United States), with a significance threshold set at p < 0.05.

Data Collection Site

Data was collected within the premises of GVPIHC&MT in Visakhapatnam, ensuring a controlled environment conducive to accurate data recording.

## Results

Among the 105 cataract patients, 55 (52.38%) were aged above 60 years, and 48 (38.10%) were aged between 30 and 60 years. Only two (1.90%) were below 30 years. Females constituted 58 (55.24%) of the participants, slightly outnumbering males at 47 (44.76%). Most patients belonged to the middle socioeconomic class with 72 (68.57%), fewer from the low (19, 18.10%) and high (14, 13.33%) classes. In terms of residential area, 43 (40.95%) were from urban areas, 36 (34.29%) from semi-urban regions, and 26 (24.76%) from rural areas (Table 1). 

**Table 1:** Demographic characteristics of cataract patients

Category	Variables	Frequency	Percentage
Age	Below 30	2	1.90%
	30-60 years	48	45.71%
	Above 60	55	52.38%
Gender	Female	58	55.24%
	Male	47	44.76%
Socioeconomic status	High	14	13.33%
	Low	19	18.10%
	Middle	72	68.57%
Residential area	Rural	26	24.76%
	Semi-urban	36	34.29%
	Urban	43	40.95%

Systemic comorbidities were prevalent among the study participants, with 43 (40.95%) having hypertension and 35 (33.33%) diabetes mellitus. Other conditions reported were less common, including asthma in 3 (2.86%), thyroid disorders in 2 (1.90%), and cardiovascular diseases in two (1.90%). Table 2 shows the prevalence of systemic comorbidities among the participants.

**Table 2:** Clinical characteristics of cataract

Category	Variables	Frequency	Percentage
Eye(s) affected	Both Eees	16	15.24%
	Left eye	40	38.10%
	Right eye	49	46.67%
Cataract type	Nuclear	16	15.24%
	Cortical	3	2.86%
	Posterior subcapsular	12	11.43%
	Mixed	74	70.48%
Duration of symptoms	Since 24 months or more	17	16.19%
	Since 12 months	52	49.52%
	Since 6 months	25	23.81%
	Since 2 months or less	11	10.48%
Cataract severity grading	Grade I	29	27.62%
	Grade II	24	22.86%
	Grade III	14	13.33%
	Grade IV	38	36.19%

The cataract cases primarily affected the right eye in 49 (46.67%) participants and the left eye in 40 (38.10%), while 16 (15.24%) cases involved both eyes. Mixed cataracts were the most frequent type observed in 74 (70.48%) of cases, followed by nuclear cataracts in 16 (15.24%), posterior subcapsular in 12 (11.43%), and cortical cataracts in 3 (2.86%). Regarding symptom duration, 17 (16.19%) participants reported symptoms for 24 months or more, 52 (49.52%) for 12 months, 25 (23.81%) for 6 months, and 11 (10.48%) for 2 months or less. Cataract severity was varied, with 38 (36.19%) cases categorized as Grade IV, 29 (27.62%) as Grade I, 24 (22.86%) as Grade II, and 14 (13.33%) as Grade III. Table 3 shows the clinical characteristics of cataract in the study participants.

**Table 3:** Association of comorbidities with cataract severity Pearson's chi-squared test; p < 0.001: highly significant; p < 0.05: significant

Category	Variable	Grade I count (%)	Grade II count (%)	Grade III count (%)	Grade IV count (%)	Total count	Chi-square value	p-value
Hypertension	No	16 (25.81%)	19 (30.65%)	11 (17.74%)	16 (25.81%)	62	10.91	0.01
	Yes	13 (30.23%)	5 (11.63%)	3 (6.98%)	22 (51.16%)	43		
Diabetes mellitus	No	22 (31.43%)	20 (28.57%)	9 (12.86%)	19 (27.14%)	70	8.88	0.03
	Yes	7 (20.00%)	4 (11.43%)	5 (14.29%)	19 (54.29%)	35		
Asthma	No	26 (25.49%)	24 (23.53%)	14 (13.73%)	38 (37.25%)	102	8.09	0.04
	Yes	3 (100.00%)	0 (0.00%)	0 (0.00%)	0 (0.00%)	3		
Thyroid	No	28 (27.18%)	23 (22.33%)	14 (13.59%)	38 (36.89%)	103	2.03	0.56
	Yes	1 (50.00%)	1 (50.00%)	0 (0.00%)	0 (0.00%)	2		
Cardiovascular diseases	No	29 (28.16%)	24 (23.30%)	14 (13.59%)	36 (34.95%)	103	3.59	0.31
	Yes	0 (0.00%)	0 (0.00%)	0 (0.00%)	2 (100.00%)	2		

The association between systemic comorbidities and cataract severity was analyzed using the chi-square test. Key findings are as follows: A significant association was found between hypertension and cataract severity (p = 0.01). Among non-hypertensive participants, 16 (25.81%), 19 (30.65%), 11 (17.74%), and 16 (25.81%) had Grade I, II, III, and IV cataract cases, respectively. In contrast, participants with hypertension had a higher proportion of Grade IV cataracts, with 22 cases (51.16%), and fewer Grade I cases, with 13 (30.23%).

A significant association was also observed between diabetes mellitus and cataract severity (p = 0.03). Non-diabetic participants had a fairly even distribution across severity levels, whereas diabetic participants showed a higher proportion of Grade IV cataracts, with 19 cases (54.29%), and fewer Grade I and Grade II cataracts, with seven cases (20.00%) and four cases (11.43%), respectively.

Although the association between asthma and cataract severity appeared statistically significant (p = 0.04), only three participants had asthma, and all had Grade I cataracts. In contrast, cataract severity among non-asthmatic participants varied across all grades (I to IV). Due to the small sample size, this finding should be interpreted cautiously.

No significant association was observed between thyroid disorders and cataract severity (p = 0.56). Only two participants had thyroid disorders, presenting with Grade I or Grade II cataracts, and the severity distribution was similar among those without thyroid disorders.

Similarly, cardiovascular disease (CVD) showed no statistically significant association with cataract severity (p = 0.31). Both participants with CVD had Grade IV cataracts, whereas cataract severity among non-CVD participants ranged from Grade I to Grade IV. However, the limited number of CVD cases restricts the reliability of this observation.

## Discussion

Our study revealed a higher prevalence of cataracts in older adults, with 52.38% aged above 60 years and 45.71% in the 30-60 years group. These findings align with Haykin et al. [5], who reported 60.9% of cataracts in the 60-70 years group and 27.3% in the 50-60 years group. Similarly, Attada et al. [6] observed 37% in the 51-60 years group and 29.1% in the 61-70 years group. Sharma et al. [7] noted 36.8% in the 61-70 years range, emphasizing age-related metabolic and structural changes as key contributors. These patterns highlight the need for targeted screening programs for older adults.

Females constituted 55.24% of our study population, indicating a slight female predominance. Similar findings were reported by Attada et al. [6] (60.5%). Sharma et al. [7] also observed a female dominance of 58%, citing increased longevity and limited healthcare access as contributing factors. However, some studies, such as Haykin et al. [5], showed higher male prevalence in the 70-80 years group, suggesting gender distribution may vary with age.

In our study, 68.57% belonged to the middle class, while 18.10% and 13.33% were from low and high SES, respectively. Studies by Sarkar et al. [8] (80%) and Sharma et al. [7] (70%) showed higher cataract prevalence in lower SES groups, reflecting disparities in healthcare access and awareness. Haykin et al. [5] reported severe cataracts in rural, low-SES populations, underscoring the impact of economic barriers on disease progression.

Most participants resided in urban (40.95%) and semi-urban (34.29%) areas, with 24.76% from rural regions. However, Mahajan et al. [9] (89.6%) reported higher cataract prevalence and severity in rural populations due to delayed diagnosis, higher UV exposure, and poor healthcare access.

In our study, 46.67% had cataracts in the right eye, 38.10% in the left eye, and 15.24% had bilateral involvement. Similar trends were observed by Gaffer et al. [10] (87% unilateral) and Sharma et al. [7] (90.3%). Haykin et al. [5] also reported a predominance of unilateral cataracts, suggesting progression often begins in one eye before affecting the other.

Nearly half (49.52%) reported symptoms for 12 months, 23.81% for six months, and 16.19% for 24 months or more. Attada et al. [6] showed shorter waiting times, with 40% seeking treatment within one year. In contrast, Chakrabarty et al. [11] reported that 45% delayed treatment for more than five years, leading to advanced cataract stages.

Mixed cataracts dominated (70.48%), followed by nuclear (15.24%), posterior subcapsular (11.43%), and cortical (2.86%). These findings differ slightly from Sharma et al. [7], where immature senile cataracts (64.6%) were most common, followed by posterior subcapsular (15.3%). Attada et al. [6] (62% immature senile) and Sarkar et al. [8] (65.2% nuclear) showed similar patterns. Sultana et al. [12] found posterior subcapsular cataracts more common in diabetic patients (27%), emphasizing the role of systemic diseases.

Hypertension was present in 40.95% of our patients, with a significant association (p = 0.0129) with cataract severity. Similar findings by Attada et al. [6] (38.3%) and Haykin et al. [5] (50%) highlight the role of hypertension in accelerating cataract development. Sarkar et al. [8] reported that hypertensive patients had 1.27 times higher odds of developing nuclear cataracts (p < 0.001) and 1.32 times higher odds of cortical cataracts, emphasizing vascular dysfunction as a risk factor.

In our study, 33.33% had diabetes, with a significant correlation with severity (p = 0.0308). Erşekerci et al. [13] reported 36%, and Sarkar et al. [8] found 75.9% of cataract patients with diabetes. Attada et al. [6] noted 29.2% with severe cataracts, while Gaffer et al. [10] found a faster progression in diabetic patients (p = 0.0476). These results reinforce diabetes as a major risk factor, leading to earlier onset and faster disease progression.

Asthma was reported in 2.86%, with no significant association (p = 0.0817) with cataract severity. Attada et al. [6] reported 6.8%, and Mahajan et al. [9] found 3.75% asthma prevalence but no correlation with severity. While corticosteroid use in asthma may accelerate cataract formation, our findings align with previous studies showing minimal impact.

Thyroid disorders were present in 1.90% of patients, with no significant association (p = 0.1572). Mahajan et al. [9] (1.87%) and Attada et al. [6] (1.2%) reported similar trends, linking hypothyroidism to early-onset cataracts due to metabolic disruptions but without direct severity associations.

CVD was identified in 1.90% of participants, with no significant correlation (p = 0.1494). Prakash et al. [14] (0.7%) and Chakrabarty et al. [11] (10.41%) found low prevalence and weak associations with severity, suggesting CVD may contribute indirectly through systemic vascular effects.

Hypertension showed a significant association with severe and very severe cataracts (p = 0.0129). Patients with hypertension were 1.32 times more likely to develop cortical cataracts, as shown by Sarkar et al. [8]. Similar findings by Jacob et al. [15] indicated hypertensive patients were at greater risk (p < 0.001), emphasizing the need for cardiovascular management to delay cataract progression.

Diabetes also showed a significant association (p = 0.0308) with severity. Sarkar et al. [8] observed increased odds (1.12 times for nuclear, 1.92 times for mature senile) of developing advanced cataracts in diabetics. Gaffer et al. [10] (p = 0.0476) and Jacob et al. [15] (p < 0.001) confirmed faster progression, suggesting aggressive glycemic control may reduce cataract severity.

No significant associations were found between asthma (p = 0.0817), thyroid disorders (p = 0.1572), or CVD (p = 0.1494) and cataract severity in our study, consistent with findings by Attada et al. [6] and Mahajan et al. [9]. This underscores the limited role of these conditions compared to hypertension and diabetes.

Limitations

Our study provides valuable insights into the prevalence and risk factors of cataracts however, certain limitations must be acknowledged. As a single-center, hospital-based study conducted over a relatively brief period, the findings may not be fully generalizable to broader populations, particularly rural and underserved communities. The overall sample size, while adequate for primary analyses, limits statistical power, especially for specific subgroup assessments. The cross-sectional design restricts the ability to establish causal relationships between systemic diseases and cataract progression. Additionally, reliance on self-reported symptom duration introduces potential recall bias. Detailed biochemical measurements such as blood pressure and blood glucose levels were not systematically recorded, possibly affecting diagnostic precision. Furthermore, initial statistical analyses did not fully account for the ordinal nature of cataract severity, although subsequent corrections were made by recommending appropriate ordinal tests. Photographic documentation of cataract grading based on LOCS III criteria was also absent, potentially affecting reproducibility. Despite these limitations, our findings align with existing literature and reinforce the significant role of aging, socioeconomic factors specific to Visakhapatnam, and systemic conditions such as hypertension and diabetes mellitus in cataract development. Future multicenter, longitudinal studies with larger cohorts and detailed methodologies can further validate and expand upon these findings.

## Conclusions

Our findings confirm that age, gender, and socioeconomic factors influence cataract prevalence, with hypertension and diabetes significantly impacting severity. Compared to previous studies, our results highlight the importance of early screening and management of systemic comorbidities to reduce the burden of severe cataracts. Addressing disparities in healthcare access, particularly in rural areas and low-SES groups, is critical to improving outcomes. Further longitudinal studies are recommended to assess causality and progression patterns over time.

## Figures and Tables

**Figure 1:**
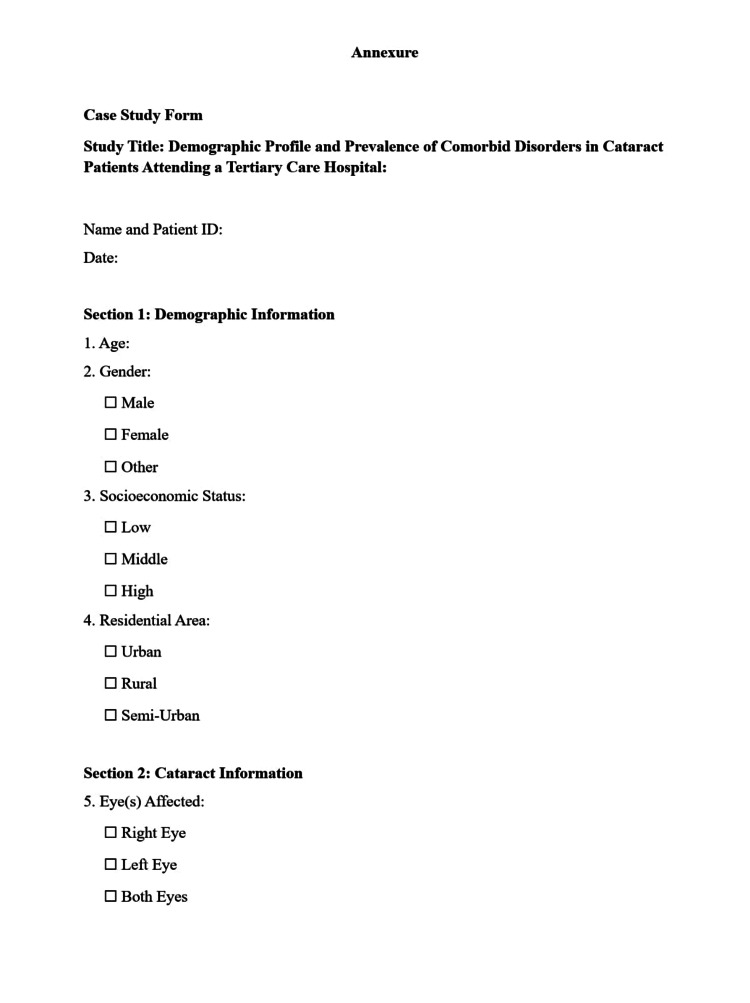
Case study proforma

**Figure 2:**
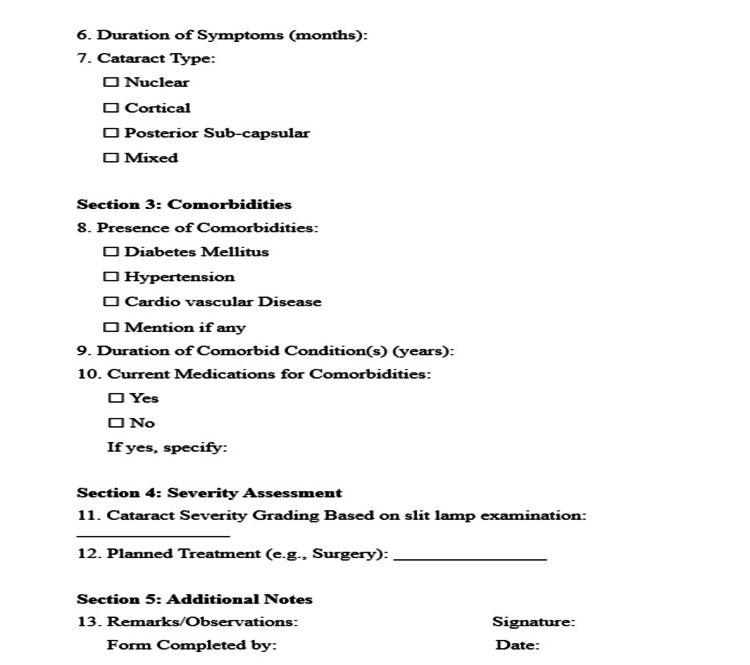
Case study proforma (Contd.)
